# Supplementary material for: Untangling the network effects of productivity and prominence among scientists
Source: Nat Commun. 2022 Aug 20;13:4907. doi: 10.1038/s41467-022-32604-6 (PMC9392727; doi:10.1038/s41467-022-32604-6)
Supplement: Supplementary file 1 — Supplementary Information [file 41467_2022_32604_MOESM1_ESM.pdf]

Supplementary Information for Untangling the network effects of productivity and prominence among scientists

Weihua Li,[1,2,3,4*] Sam Zhang,[5] Zhiming Zheng,[1,2,3,4] Skyler J. Cranmer,[6] Aaron Clauset[7,8,9,*]

[1]*LMIB, NLSDE, BDBC, and Institute of Artificial Intelligence, Beihang University, Beijing 100191, China*

[2]*Peng Cheng Laboratory, Shenzhen 518055, China*

[3]*Zhong Guan Cun Laboratory, Beijing 100080, China*

[4]*Beijing Academy of Blockchain and Edge Computing, Beijing 100080, China*

[5]*Department of Applied Mathematics, University of Colorado Boulder, Boulder, CO 80309, USA*

[6]*Department of Political Science, The Ohio State University, Columbus, OH 43210, USA*

[7]*Department of Computer Science, University of Colorado, Boulder, CO 80309, USA*

[8]*BioFrontiers Institute, University of Colorado, Boulder, CO 80309, USA*

[9]*Santa Fe Institute, Santa Fe, NM 87501, USA*

** Correspondence should be addressed to W.L. (email: weihuali@buaa.edu.cn) or to A.C. (email: aaron.clauset@colorado.edu)*

Supplementary Note 1 Selection of research fields

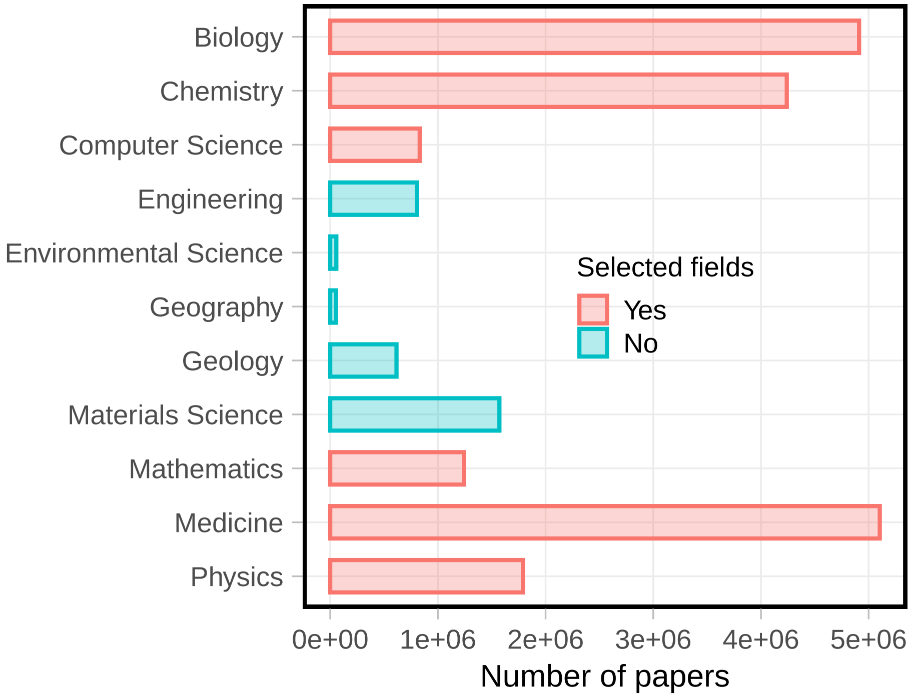

Supplementary Figure 1. **Total publication counts for major research fields.** We show the number of publications for all level 0 fields in STEM fields. The red fields are selected to estimate the latent variable models, which publish the majority of research articles.

Supplementary Note 2 Estimation of latent variable models

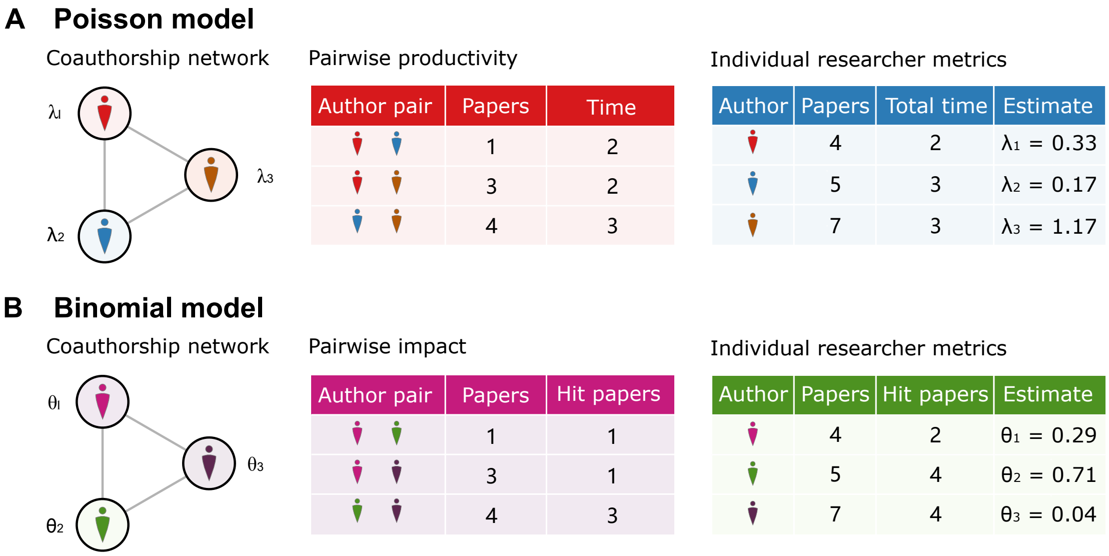

Supplementary Figure 2. **Illustrative example to explain latent variable models. a**, on average, the annual publication rate for authors 1, 2, 3 are 2, 1.67 and 2.33, respectively, among which author 3 is the most productive and our Poisson model suggests that she should be given the most credit for the productivity (latent variable $\lambda = 1.17$). **b**, on average, the raw probability to publish a hit paper for authors 1, 2, 3 are 0.5, 0.8 and 0.57, respectively, among which author 2 has the highest hit paper rate and our binomial model suggests that she should be given the most credit for attracting research impact (latent variable $\theta_2 = 0.71$). We provide R code for computing these illustrative models in the appendix.

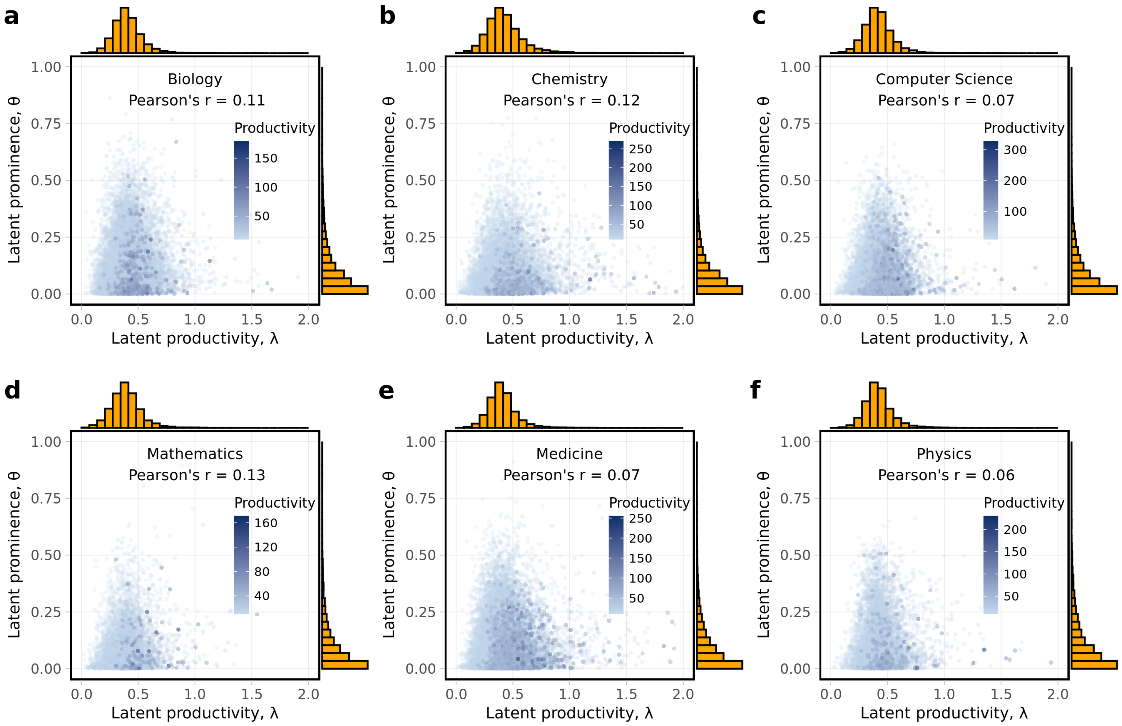

Supplementary Figure 3. **The distributions of latent variables λ and θ in 2017 for all authors with $\theta > 0.001$, for each of the six individual disciplines.**

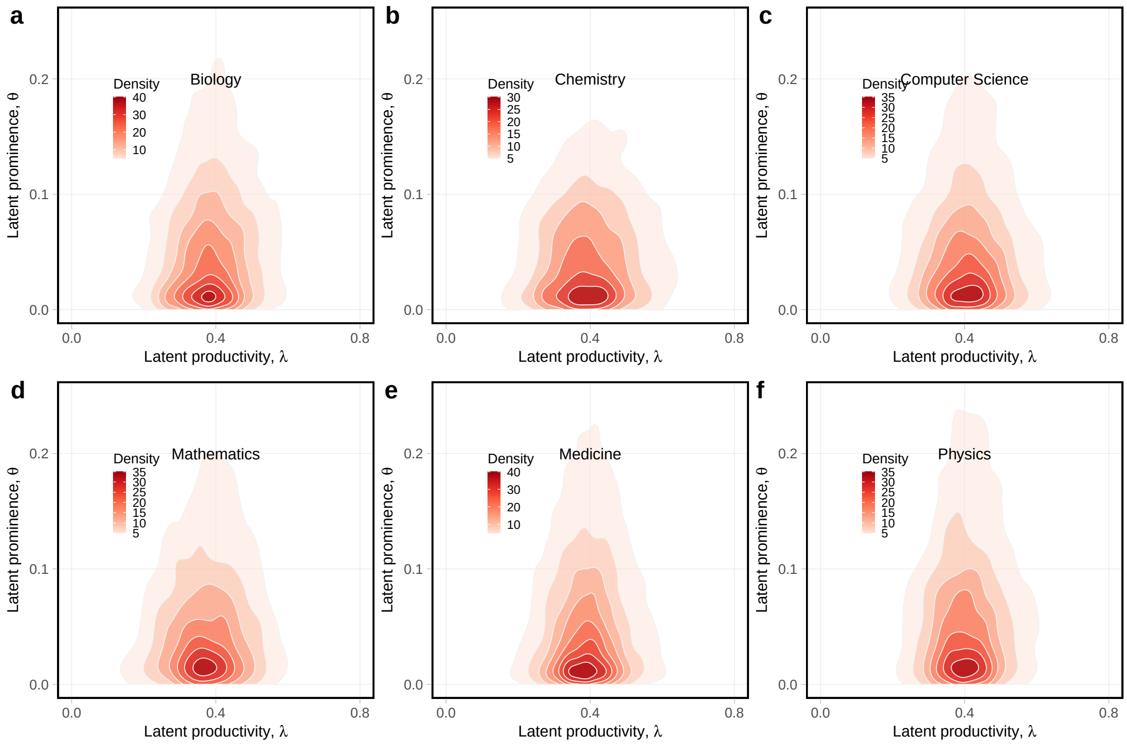

Supplementary Figure 4. **Contour plots of the distributions of latent variables λ and θ in 2017 for all authors with $\theta > 0.001$, for each of the six individual disciplines.**

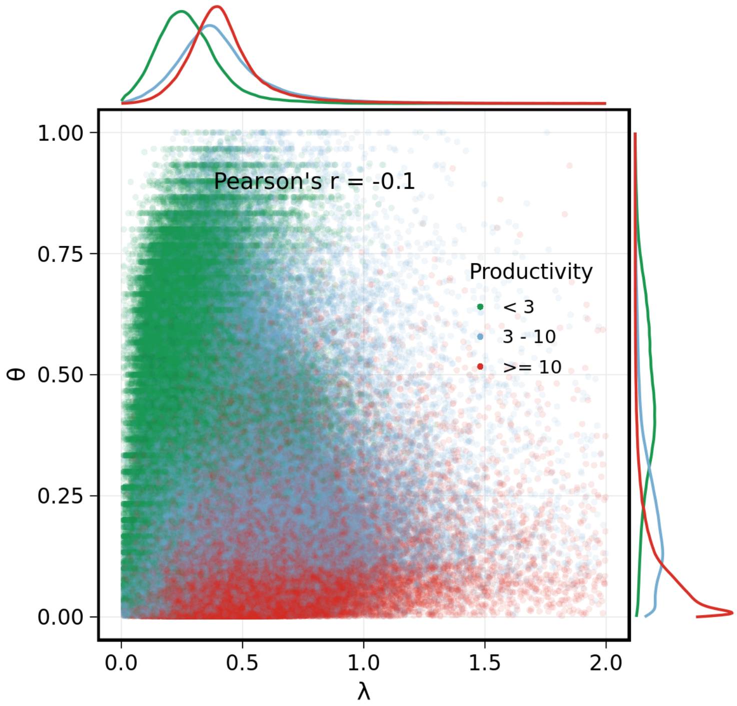

Supplementary Figure 5. **The distributions of latent variables λ and θ in 2017 based on author productivity.** λ follows a normal distribution, while θ is heavy-tailed. The correlation coefficient between λ and θ is weak.

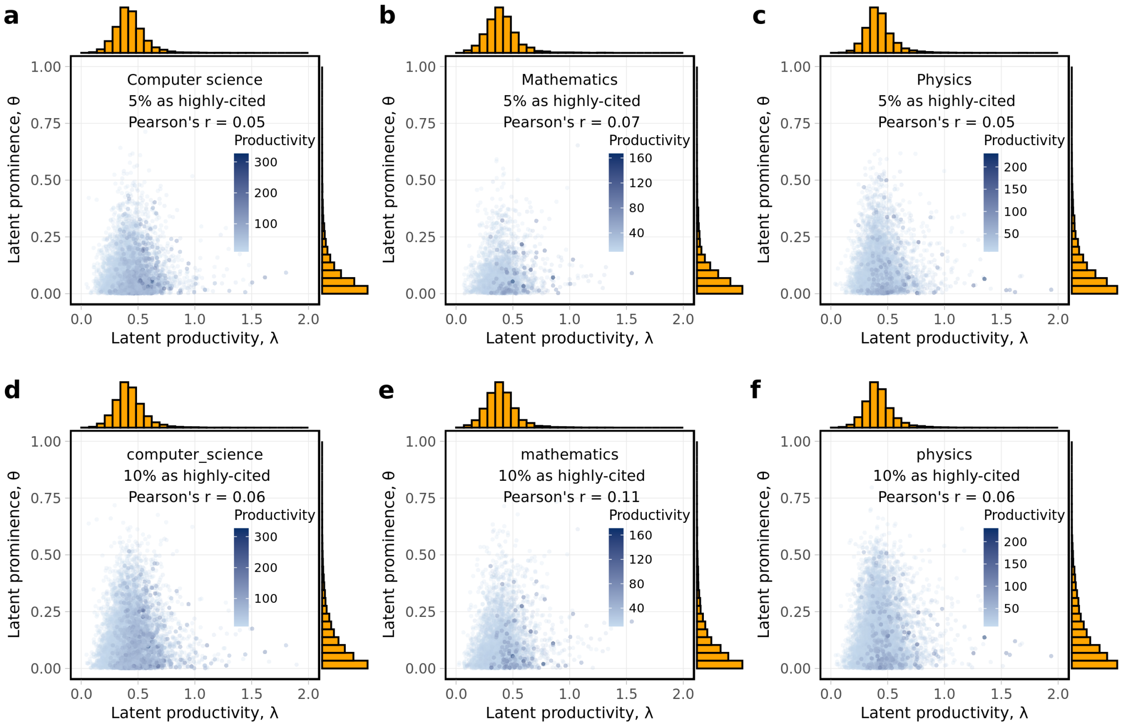

Supplementary Figure 6. **Replicating latent prominence model.** We re-compute the θ values of mid-career researchers for **a** computer science, **b** mathematics, and **c** physics by defining highly-cited papers as those receiving the the **a-c** upper 5th and **d-f** 10th percentile citations two years after publication.

Supplementary Note 3 Stability tests of the latent variables

a Empirical data

Author	5th year	15th year
1	high	high
2	medium	medium
3	medium	low
4	low	medium
5	low	low
6	low	low

b Null model

Author	5th year	15th year (randomized)
1	high	low
2	medium	low
3	medium	medium
4	low	low
5	low	high
6	low	medium

Supplementary Figure 7. **Illustrative plot to explain the randomized null model when we measure the stability of latent parameters.** In the null model, for latent variables λ and θ we randomly shuffle the later year positions of researchers using 100 replications.

To examine the career-wise stability of latent variables λ and θ, we group authors based on their percentile rankings of the latent variables for a given year and field. Considering the heavy-tail nature of distribution of θ, we classify authors with the top 10% latent parameter values as high, $10-40\%$ as medium and the bottom 60% as low for both λ and θ (Fig. Supplementary Figure 8 **a** and **c**). We find that both latent variables are relatively stable over the career trajectory of a researcher, measured by the probability that a given author remains in the same percentile group after 10 years, compared to the values predicted by a fully randomized null model (Fig. S5). This effect is particularly strong for researchers with high latent parameter values. Authors with high 5th year λ/θ values are very unlikely to fall to the low percentile group in the 15th year, with a probability of 19.2% and 2.2% for λ and θ, respectively. Authors with low 5th year λ/θ values are very likely to remain there in the 15th year, and their probability of getting high mid-career parameter values is merely 4.0% for λ and 2.9% for θ. The stability of latent variables provides extra evidence and insight for the reported long-term academic stratification of scientific careers since the early stage.

We also use the solo-authored papers among mid-career researchers to assess the efficacy of

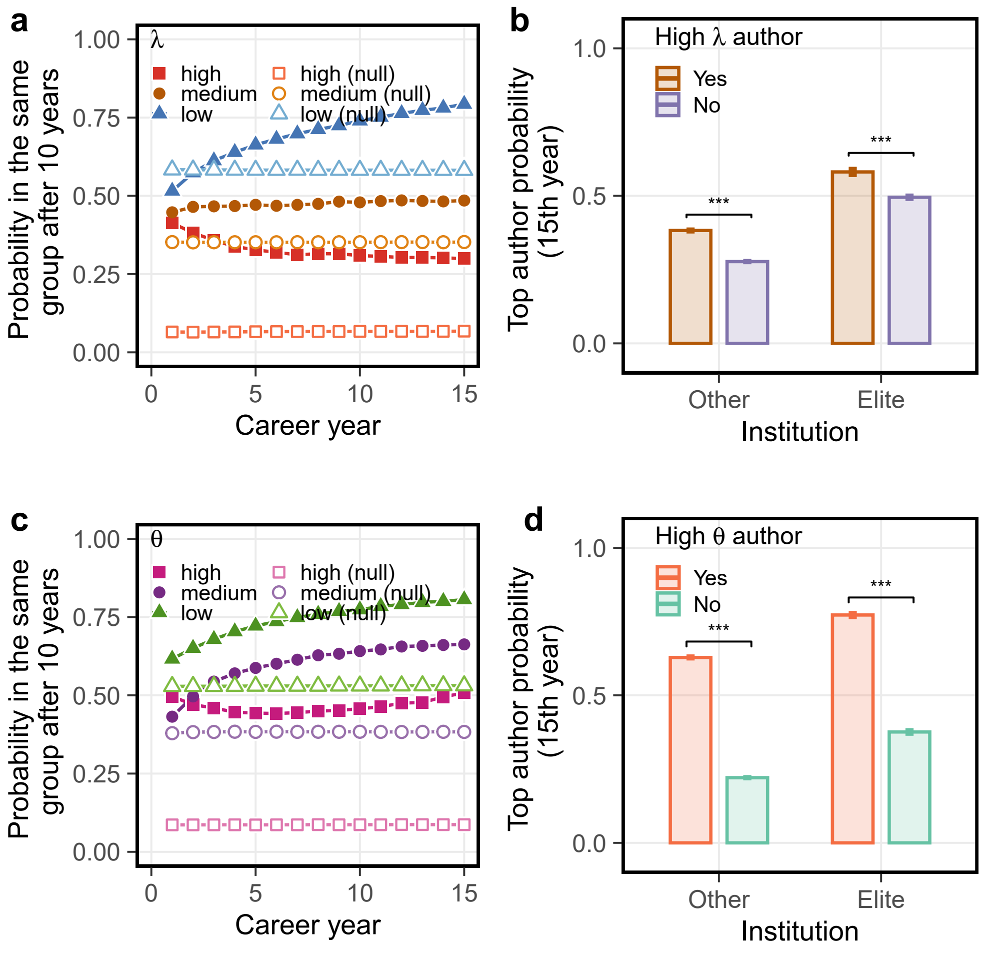

Supplementary Figure 8. **Career-wise stability of the latent variables λ and θ.** The probability that an author remains in the same percentile group after 10 years is plotted for λ (**a**), and θ (**c**). Solid dots indicate empirical probabilities, while hollow dots show the average probability in a null model in which we randomly permute real values for the later years in 100 replications (Fig. Supplementary Figure 7). We also use the selected authors' 5th year data and the estimated latent variables λ and θ to predict the probability of becoming top authors in the 15th year. Authors with high early-career parameter value of λ (**b**) or θ (**d**) have higher probability of becoming top author in the mid career. Researchers with early high λ, n for $\text{yes} = 107,528$; $\text{no} = 90,674$. Researchers with early high θ, n for $\text{yes} = 65,985$; $\text{no} = 132,217$. Error bars in (**b** and **d**) indicate mean $\pm$ 1.96 SEM. (***$p < 0.001$; **$p < 0.01$; *$p < 0.05$).

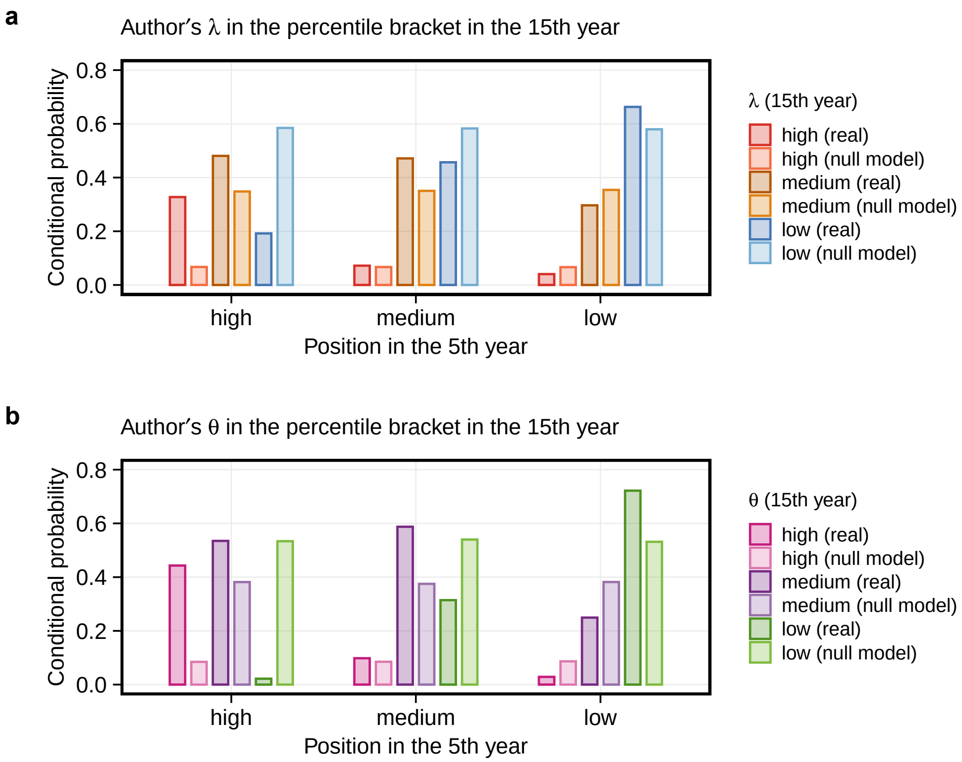

Supplementary Figure 9. **Summary statistics for the probability that an author's λ and θ values in the percentile brackets in the 15th career year conditional on their position in the 5th year, for both real values and values in the fully randomized null model.** The results show that, for researchers with high λ or θ values in the 5th year, the probability to keep having high λ or θ in the 15th year is markedly higher than that predicted by a randomized null model. If the researchers have low λ or θ values in the early-career, then the chances that they will have high latent parameter values in the mid-career is particularly small, compared to the prediction of the randomized null model.

θ. Among these mid-career researchers, 90,583 of them published at least one solo-authored paper. There are 48,484 researchers with negligible estimated values of $\theta < 10^{-3}$, and 84.8% (41,102) of them published no highly-cited solo-authored paper up to the mid-career. To test if the prominence model (θ) better predicts researchers' impact, we use a random null model to estimate a baseline of publishing highly-cited work. The null model randomly reshuffles solo-authored papers among authors, and we find that 79.0% of researchers with $\theta < 10^{-3}$ published no highly-cited solo-authored papers, 5.8% lower than the empirical percentage. This suggests that low estimated θ value moderately increases the precision in predicting whether researchers have highly-cited solo-authored papers or not.

We show how the latent parameters and the prestige of academic environment of junior researchers in the 5th year predict academic success in the 15th year in Fig. Supplementary Figure 8. Elite institutions are defined as those among the top 50 research institutions for a given field, and are used to compare with other ordinary institutions to exhibit the effect of research environment on academic careers. Top authors are researchers whose total number of citations is among the top 5% of all active researchers for a given year and field. The author has high latent parameter values of λ/θ if he/she is in the upper 20th percentile among all 5th year researchers for a given year and field. In Fig. Supplementary Figure 8 **b** and **d**, we see that authors that have high early-career latent parameter λ/θ values have an significantly increased chance to become a top scientist in the mid-career. Higher early-career θ value foresees a more prominent mid-career prospect for junior researchers, suggesting that individual impact provides more boosting effect for scientific careers.

The performance of researchers is driven by a mixture of complicated factors, which usually include personal scientific capability, age, collaborators, research environment, and more. We use matched-pairs experiments to examine whether the predictive patterns of latent variables λ and θ persist after accounting for these factors [1]. We do so by randomly selecting author pairs

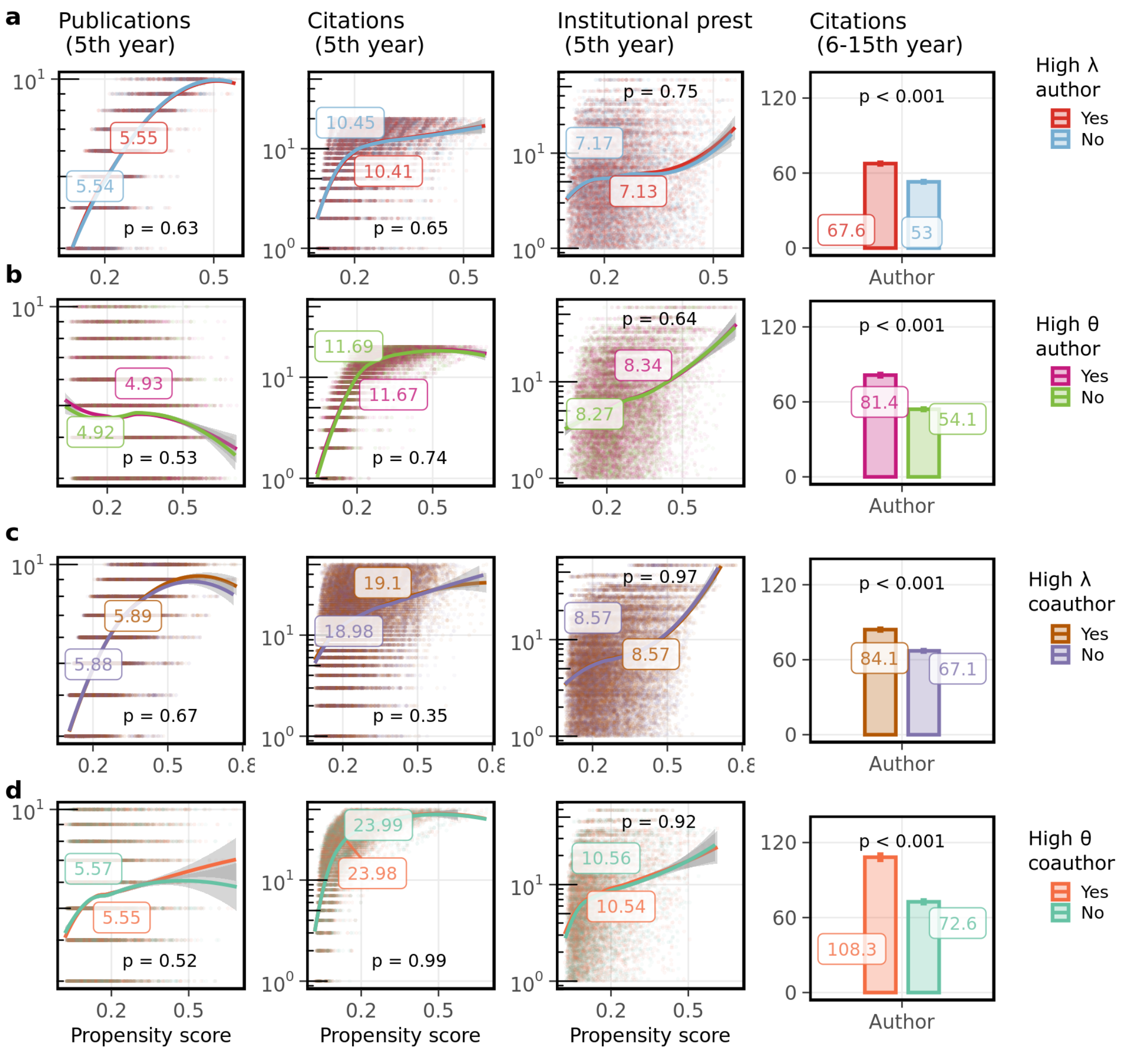

Supplementary Figure 10. **Matched-pair analyses of mid-career performance based on early-career latent variables λ and θ.** Left three columns: propensity score plots of the matched pairs of authors with each dot representing an individual author. Shaded areas represent 95% confidence intervals. Right column: average number of citations for the matched groups of authors in the 15th year, excluding those from papers published in the first 5 career years. The researcher's λ (**a**) or θ (**b**) is high if it is in the upper 20% of all selected authors for a given year and field. If at least one of the early-career collaborators has high λ (**c**) or θ (**d**) in the upper 10% of all active authors for a given year and field. Researchers with early high λ, n for $\text{yes} = 12,130$; $\text{no} = 12,130$. Researchers with early high θ, n for $\text{yes} = 20,251$; $\text{no} = 20,251$. Researchers with early high λ coauthors, n for $\text{yes} = 12,131$; $\text{no} = 12,131$. Researchers with early high θ coauthors, n for $\text{yes} = 8,637$; $\text{no} = 8,637$. Two-sided t-test were used for comparisons. Error bands (1st, 2nd, and 3rd panels) indicate mean $\pm$ 1.96 SEM. Error bars (4th panel) indicate mean $\pm$ 1.96 SEM.

after controlling for discipline and their 5th year productivity, citations and prestige of institutions (Fig. Supplementary Figure 10). To exclude outliers and obtain a more robust matching results, we retain authors that have no more than 20 citations and up to 10 papers in the 5th year. In the matched-pair experiments, no statistically difference of the early-career covariates is found between the treatment and control groups for both latent variables λ and θ. However, authors with high early-career latent variable λ/θ values still significantly outperform the others in mid-career. The authors with high early-career λ gets 27.6% more citations than authors in the control group, in $12,130$ matched pairs of authors. This competitive advantage rises to 50.7% for authors with high early-career θ in $12,131$ matched pairs.

Similarly, we investigate the early-career coauthor effects in Fig. Supplementary Figure 10 **c** and **d**, by setting the citation upper bound to 50 and accounting for coauthors that have at least 3 papers. No statistically difference of the early-career covariates is found between the treatment and control groups for both latent variables λ and θ of their collaborators. Yet, authors with high λ coauthors significantly outperform the others by 25.3% in mid-career in $20,251$ matched pairs of authors, and this impact premium increases to 49.1% for authors with high θ coauthors in $8,637$ matched pairs [2]. In tables Supplementary Table 1, Supplementary Table 2, Supplementary Table 3, Supplementary Table 4, we present detailed disciplinary analyses for each of the author/coauthor and λ/θ effects, and the results further enhance our findings in the aggregate data of individual authors.

In Supplementary Table 1, the matched-pairs experiment, we assign a researcher to the treatment group if his/her λ value in the early career is among the upper 10th percentile for a given year and field, otherwise he/she is assigned to the control group. We consider authors with at least 3 but no more than 10 publications, and no more than 20 citations in the 5th year. This allows us to eliminate less productive early-career researchers whose latent parameter estimates are less reli-

	Biology			Chemistry			Computer Science		
No. authors	12,996			12,580			7,928		
No. matched pairs	2,602			2,515			1,591		
	Treat	Control	p	Treat	Control	p	Treat	Control	p
Inst. prestige (5th yr)	6.32	6.23	0.74	6.07	6.12	0.86	6.71	6.83	0.66
	(0.19)	(0.19)		(0.19)	(0.23)		(0.18)	(0.20)	
Productivity (5th yr)	4.97	4.96	0.83	5.70	5.70	0.92	5.85	5.80	0.5
	(0.03)	(0.03)		(0.04)	(0.04)		(0.05)	(0.05)	
Citations (5th yr)	11.62	11.67	0.74	10.52	10.48	0.79	10.20	10.32	0.56
	(0.11)	(0.11)		(0.11)	(0.11)		(0.14)	(0.14)	
First year	1992.75	1992.87	0.53	1993.10	1993.10	0.97	1997.07	1996.78	0.13
	(0.14)	(0.14)		(0.14)	(0.14)		(0.14)	(0.14)	
Productivity (6-15th yr)	12.08	13.20	***	13.99	15.09	**	18.60	19.60	0.06
	(0.14)	(0.17)		(0.23)	(0.26)		(0.36)	(0.39)	
Citations (6-15th yr)	76.38	53.32	***	60.37	43.22	***	60.71	51.17	*
	(2.03)	(1.64)		(2.43)	(1.61)		(2.88)	(2.79)	

	Mathematics			Medicine			Physics		
No. authors	4,456			16,096			6,516		
No. matched pairs	896			3,220			1,306		
	Treat	Control	p	Treat	Control	p	Treat	Control	p
Inst. prestige (5th yr)	5.95	6.53	*	8.99	9.08	0.76	7.50	7.20	0.52
	(0.20)	(0.21)		(0.20)	(0.20)		(0.33)	(0.33)	
Productivity (5th yr)	5.60	5.56	0.68	5.66	5.65	0.89	5.76	5.76	0.98
	(0.06)	(0.06)		(0.03)	(0.04)		(0.05)	(0.05)	
Citations (5th yr)	10.05	10.02	0.91	9.86	9.91	0.71	9.68	9.71	0.88
	(0.18)	(0.18)		(0.10)	(0.10)		(0.16)	(0.16)	
First year	1994.58	1994.56	0.96	1994.12	1993.98	0.39	1993.07	1993.07	0.98
	(0.21)	(0.22)		(0.12)	(0.12)		(0.18)	(0.17)	
Productivity (6-15th yr)	12.61	12.92	0.47	15.26	15.67	0.19	12.70	12.79	0.79
	(0.28)	(0.33)		(0.21)	(0.23)		(0.23)	(0.24)	
Citations (6-15th yr)	47.72	40.47	0.08	78.95	67.40	***	57.92	46.13	**
	(3.31)	(2.42)		(2.17)	(2.35)		(2.97)	(2.58)	

Supplementary Table 1. **Matched pair analysis to treat if the researcher has high λ in the 5th year.** (*: $p < 0.05$; **: $p < 0.01$; ***: $p < 0.001$; NS: not significant). Numbers in brackets denote standard errors.

able, and also exclude super productive researchers that are hard to find close matches in the other group. For each field, matches are constructed by randomly selecting pairs of researchers from both groups that has similar attribute scores of institutional prestige, number of papers, number of citations, and the start year. We present the mean values of attributes for the matched pairs of researchers, and p-values of t-tests to show whether the mean values of both samples are significantly different. The matching results are generally good in that the early-career attribute values of selected researchers are not significantly different, except that one group of researchers have high λ and those in the other group do not have high λ in the 5th year. In the mid-career, however, while the gap of productivity is moderate or indistinguishable for most fields, in five out of six fields the high λ group receives higher average citations for their work published between 6-15th career years.

In Supplementary Table 2, the matched-pairs experiment, we assign a researcher to the treatment group if his/her θ value in the early career is among the upper 10th percentile for a given year and field, otherwise he/she is assigned to the control group. We consider authors with at least 3 but no more than 10 publications, and no more than 20 citations in the 5th year. This allows us to eliminate less productive early-career researchers whose latent parameter estimates are less reliable, and also exclude super productive researchers that are hard to find close matches in the other group. For each field, matches are constructed by randomly selecting pairs of researchers from both groups that has similar attribute scores of institutional prestige, number of papers, number of citations, and the start year. We present the mean values of attributes for the matched pairs of researchers, and p-values of t-tests to show whether the mean values of both samples are significantly different. The matching results are generally good in that the early-career attribute values of selected researchers are not significantly different, except that one group of researchers have high θ and those in the other group do not have high θ in the 5th year. In the mid-career, however, while the gap of productivity is moderate or negligible for most fields, in all six fields the high

	Biology			Chemistry			Computer Science		
No. authors	12,996			12,580			7,928		
No. matched pairs	2,604			2,517			1,589		
	Treat	Control	p	Treat	Control	p	Treat	Control	p
Inst. prestige (5th yr)	7.38	7.02	0.2	6.59	6.89	0.37	7.72	7.48	0.42
	(0.20)	(0.20)		(0.21)	(0.26)		(0.21)	(0.20)	
Productivity (5th yr)	4.52	4.48	0.28	5.03	5.00	0.54	5.31	5.33	0.85
	(0.03)	(0.03)		(0.04)	(0.04)		(0.05)	(0.05)	
Citations (5th yr)	11.55	11.67	0.44	11.24	11.20	0.78	12.03	12.07	0.82
	(0.11)	(0.11)		(0.11)	(0.11)		(0.13)	(0.13)	
First year	1992.75	1992.68	0.72	1993.09	1993.30	0.28	1997.07	1996.99	0.67
	(0.14)	(0.14)		(0.14)	(0.14)		(0.14)	(0.14)	
Productivity (6-15th yr)	12.77	12.57	0.35	15.24	14.26	**	19.70	19.01	0.22
	(0.15)	(0.15)		(0.27)	(0.23)		(0.37)	(0.42)	
Citations (6-15th yr)	84.60	60.01	***	66.14	42.27	***	81.52	52.59	***
	(2.66)	(2.11)		(2.59)	(1.80)		(4.24)	(2.69)	

	Mathematics			Medicine			Physics		
No. authors	4,456			16,096			6,516		
No. matched pairs	895			3,218			1,308		
	Treat	Control	p	Treat	Control	p	Treat	Control	p
Inst. prestige (5th yr)	6.12	6.24	0.7	10.88	10.78	0.74	9.63	9.60	0.96
	(0.20)	(0.21)		(0.22)	(0.23)		(0.36)	(0.40)	
Productivity (5th yr)	4.94	4.94	0.99	4.93	4.93	1	5.11	5.11	0.97
	(0.06)	(0.06)		(0.03)	(0.03)		(0.05)	(0.05)	
Citations (5th yr)	12.51	12.48	0.92	11.76	11.77	0.96	11.47	11.48	0.97
	(0.17)	(0.17)		(0.10)	(0.10)		(0.16)	(0.16)	
First year	1994.59	1994.60	0.96	1994.13	1994.22	0.63	1993.07	1993.19	0.64
	(0.21)	(0.21)		(0.12)	(0.12)		(0.18)	(0.18)	
Productivity (6-15th yr)	13.14	11.87	**	16.20	14.91	***	13.66	12.62	**
	(0.30)	(0.25)		(0.22)	(0.19)		(0.23)	(0.23)	
Citations (6-15th yr)	59.06	37.42	***	99.17	66.97	***	76.19	46.26	***
	(4.74)	(1.92)		(2.66)	(2.13)		(3.32)	(2.73)	

Supplementary Table 2. **Matched pair analysis to treat if the author has high θ in the 5th year.** (*: $p < 0.05$; **: $p < 0.01$; ***: $p < 0.001$; NS: not significant). Numbers in brackets denote standard errors.

θ group receives substantively higher average citations for their work published between 6-15th career years.

In Supplementary Table 3, the matched-pairs experiment, we assign a researcher to the treatment group if he/she has collaborated in the early-career with at least one coauthor who has a high λ value, published at least 3 papers, and has at least 6 years of publishing career by the time of collaboration, otherwise he/she is assigned to the control group. We consider authors with at least 3 but no more than 10 publications, and no more than 50 citations in the 5th year. This allows us to eliminate less productive early-career researchers whose latent parameter estimates are less reliable, and also exclude super productive researchers that are hard to find close matches in the other group. For each field, matches are constructed by randomly selecting pairs of researchers from both groups that has similar attribute scores of institutional prestige, number of papers, number of citations, and the start year. We present the mean values of attributes for the matched pairs of researchers, and p-values of t-tests to show whether the mean values of both samples are significantly different. The matching results are generally good in that the early-career attribute values of selected researchers are not significantly different, except that one group of researchers have high λ coauthors and those in the other group do not have high λ coauthors in the 5th year. In the mid-career, however, while the gap of productivity is moderate or negligible for most fields, in all six fields the group with high λ coauthors receives substantively higher average citations for their work published between 6-15th career years.

In Supplementary Table 4, the matched-pairs experiment, we assign a researcher to the treatment group if he/she has collaborated in the early-career with at least one coauthor who has a high θ value, published at least 3 papers, and has at least 6 years of publishing career by the time of collaboration, otherwise he/she is assigned to the control group. We consider authors with at least 3 but no more than 10 publications, and no more than 50 citations in the 5th year. This allows

	Biology			Chemistry			Computer Science		
No. authors	23,286			17,596			11,203		
No. matched pairs	4,006			3,392			2,399		
	Treat	Control	p	Treat	Control	p	Treat	Control	p
Inst. prestige (5th yr)	7.43	7.50	0.77	6.03	6.21	0.48	8.25	8.34	0.74
	(0.17)	(0.17)		(0.18)	(0.19)		(0.17)	(0.18)	
Productivity (5th yr)	5.49	5.50	0.95	6.06	6.06	0.95	6.21	6.22	0.89
	(0.03)	(0.03)		(0.04)	(0.04)		(0.04)	(0.04)	
Citations (5th yr)	23.05	22.80	0.4	17.00	16.88	0.68	19.51	19.32	0.62
	(0.21)	(0.21)		(0.21)	(0.21)		(0.27)	(0.28)	
First year	1992.75	1992.59	0.3	1993.89	1993.84	0.8	1997.08	1997.07	0.97
	(0.11)	(0.11)		(0.12)	(0.12)		(0.11)	(0.11)	
Productivity (6-15th yr)	13.35	13.17	0.34	15.73	15.16	0.07	20.76	20.21	0.24
	(0.14)	(0.14)		(0.23)	(0.22)		(0.33)	(0.33)	
Citations (6-15th yr)	94.77	74.15	***	67.25	53.49	***	81.45	64.38	***
	(2.43)	(2.21)		(2.20)	(2.07)		(3.39)	(2.45)	

	Mathematics			Medicine			Physics		
No. authors	5,798			23,606			9,453		
No. matched pairs	1,538			6,323			2,593		
	Treat	Control	p	Treat	Control	p	Treat	Control	p
Inst. prestige (5th yr)	6.12	6.29	0.45	10.93	10.77	0.48	9.64	9.47	0.66
	(0.16)	(0.16)		(0.15)	(0.16)		(0.28)	(0.28)	
Productivity (5th yr)	5.82	5.81	0.86	5.86	5.84	0.47	6.06	6.05	0.85
	(0.05)	(0.05)		(0.03)	(0.03)		(0.04)	(0.04)	
Citations (5th yr)	15.99	16.04	0.91	18.56	18.51	0.85	18.54	18.40	0.7
	(0.30)	(0.30)		(0.16)	(0.17)		(0.26)	(0.26)	
First year	1995.01	1994.98	0.91	1994.69	1994.75	0.66	1993.34	1993.22	0.51
	(0.16)	(0.16)		(0.09)	(0.09)		(0.13)	(0.13)	
Productivity (6-15th yr)	13.41	12.64	*	16.55	16.29	0.26	13.44	12.97	0.06
	(0.23)	(0.24)		(0.17)	(0.16)		(0.19)	(0.17)	
Citations (6-15th yr)	57.91	41.96	***	97.47	82.33	***	75.13	54.67	***
	(3.08)	(2.40)		(1.97)	(1.76)		(3.14)	(2.48)	

Supplementary Table 3. **Matched pair analysis to treat if the author has a high λ coauthor up to the 5th year.** (*: $p < 0.05$; **: $p < 0.01$; ***: $p < 0.001$; NS: not significant). Numbers in brackets denote standard errors.

	Biology			Chemistry			Computer Science		
No. authors	23,286			17,596			11,203		
No. matched pairs	1,984			1,591			1,076		
	Treat	Control	p	Treat	Control	p	Treat	Control	p
Inst. prestige (5th yr)	10.45	10.32	0.76	7.91	8.00	0.84	9.71	9.82	0.79
	(0.30)	(0.30)		(0.29)	(0.35)		(0.28)	(0.29)	
Productivity (5th yr)	4.92	5.01	0.13	5.65	5.65	0.94	5.97	5.96	0.87
	(0.04)	(0.04)		(0.05)	(0.05)		(0.06)	(0.06)	
Citations (5th yr)	27.70	27.89	0.64	22.71	22.72	0.98	24.30	24.26	0.94
	(0.29)	(0.30)		(0.33)	(0.33)		(0.41)	(0.41)	
First year	1992.86	1992.77	0.69	1993.62	1993.75	0.61	1997.28	1997.21	0.77
	(0.16)	(0.16)		(0.17)	(0.18)		(0.17)	(0.17)	
Productivity (6-15th yr)	13.31	13.08	0.38	15.16	14.49	0.11	20.68	19.61	0.11
	(0.18)	(0.19)		(0.32)	(0.28)		(0.46)	(0.49)	
Citations (6-15th yr)	126.74	89.36	***	78.99	56.04	***	110.16	71.14	***
	(5.19)	(3.50)		(3.75)	(2.43)		(6.56)	(5.45)	

	Mathematics			Medicine			Physics		
No. authors	5,798			23,606			9,453		
No. matched pairs	729			1,715			1,542		
	Treat	Control	p	Treat	Control	p	Treat	Control	p
Inst. prestige (5th yr)	7.13	7.20	0.84	13.52	13.82	0.52	12.25	11.99	0.65
	(0.23)	(0.25)		(0.33)	(0.34)		(0.37)	(0.45)	
Productivity (5th yr)	5.71	5.68	0.71	5.60	5.60	0.96	5.83	5.86	0.64
	(0.07)	(0.07)		(0.05)	(0.05)		(0.05)	(0.05)	
Citations (5th yr)	19.71	19.70	0.99	22.95	22.73	0.63	23.47	23.49	0.97
	(0.46)	(0.46)		(0.31)	(0.32)		(0.34)	(0.34)	
First year	1995.29	1995.40	0.73	1994.49	1994.57	0.74	1993.64	1993.70	0.8
	(0.22)	(0.23)		(0.17)	(0.17)		(0.16)	(0.17)	
Productivity (6-15th yr)	13.13	12.85	0.53	17.47	16.53	*	13.47	12.74	*
	(0.32)	(0.32)		(0.34)	(0.31)		(0.22)	(0.22)	
Citations (6-15th yr)	66.13	50.73	**	132.11	89.88	***	106.80	60.29	***
	(4.99)	(3.09)		(6.75)	(3.30)		(6.92)	(2.85)	

Supplementary Table 4. **Matched pair analysis to treat if the author has a high θ coauthor up to the 5th year.** (*: $p < 0.05$; **: $p < 0.01$; ***: $p < 0.001$; NS: not significant). Numbers in brackets denote standard errors.

us to eliminate less productive early-career researchers whose latent parameter estimates are less reliable, and also exclude super productive researchers that are hard to find close matches in the other group. For each field, matches are constructed by randomly selecting pairs of researchers from both groups that has similar attribute scores of institutional prestige, number of papers, number of citations, and the start year. We present the mean values of attributes for the matched pairs of researchers, and p-values of t-tests to show whether the mean values of both samples are significantly different. The matching results are generally good in that the early-career attribute values of selected researchers are not significantly different, except that one group of researchers have high θ coauthors and those in the other group do not have high θ coauthors in the 5th year. In the mid-career, however, while the gap of productivity is moderate or negligible for most fields, in all six fields the group with high θ coauthors receives substantively higher average citations for their work published between 6-15th career years.

Supplementary Note 4 Gender disparity

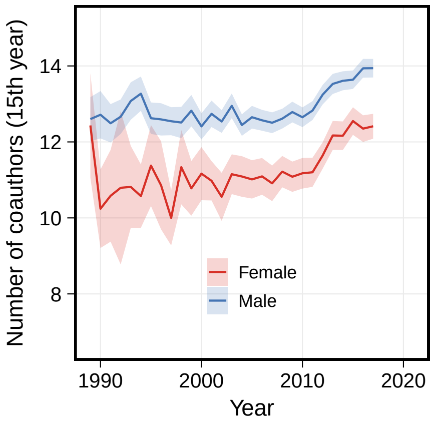

Supplementary Figure 11. **The number of collaborators for men and women in the mid-career.** The results show that women have fewer collaborators than men up to the 15th career year. Solid lines indicate mean and shaded areas indicate 95% confidence intervals.

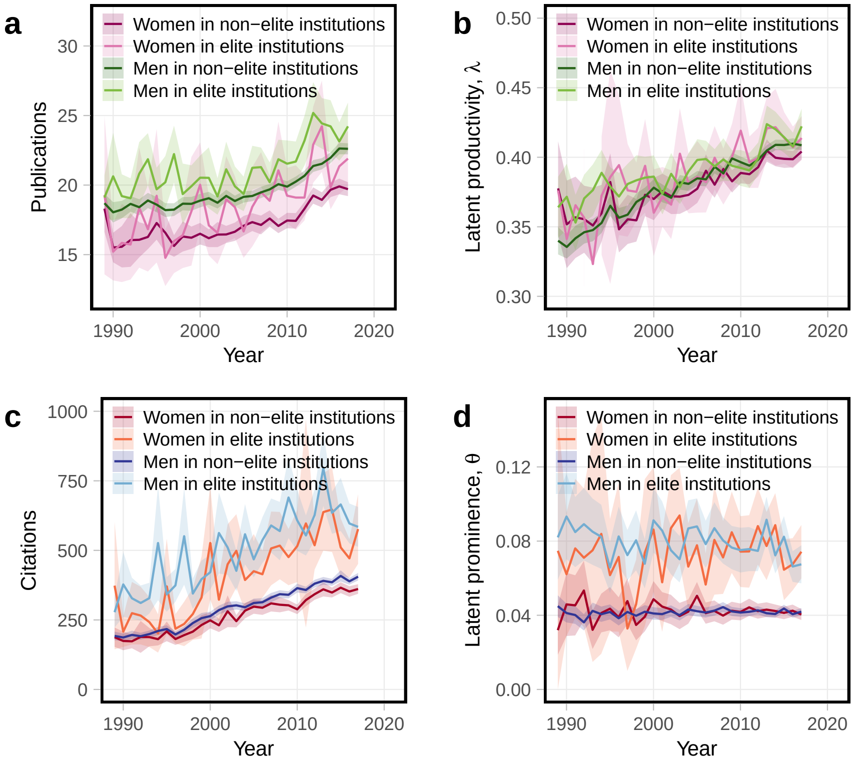

Supplementary Figure 12. **Interaction of gender and institution effects for mid-career researchers.** We test the **a** publications, **b** latent productivity λ, **c** citations, and **d** latent prominence θ for mid-career researchers by the interaction of institution and gender. Two-sided t-test for comparisons. In (**a**, **b**, **d**, and **e**), shaded areas represent 95% confidence intervals.

Supplementary Note 5 Robustness tests

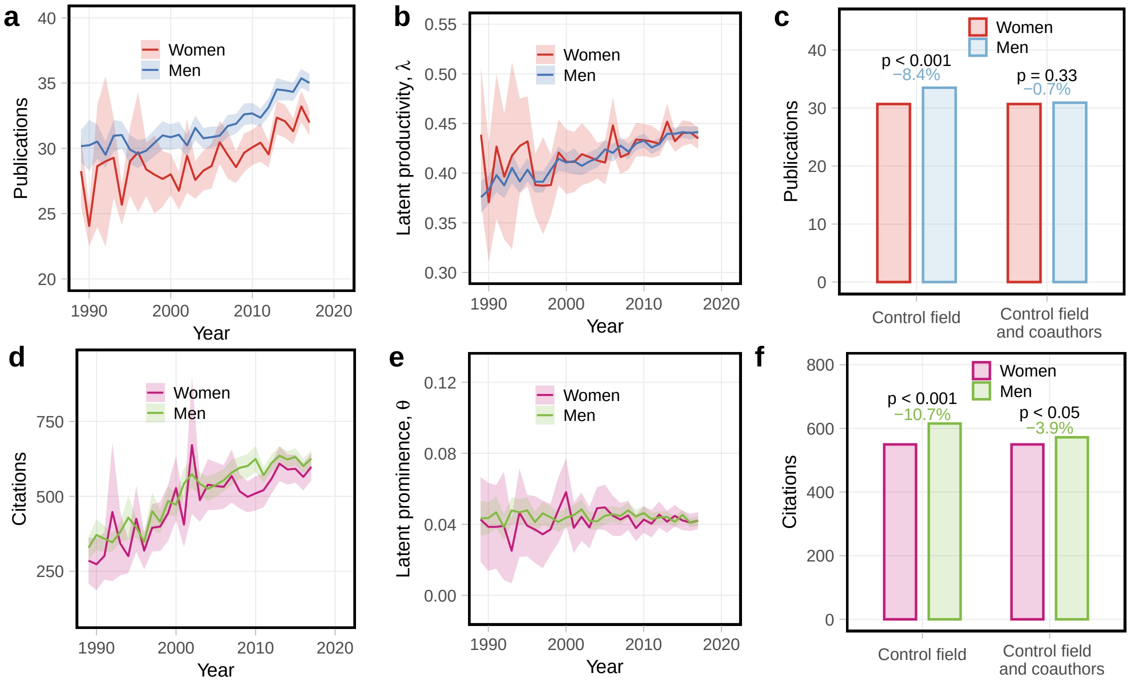

Supplementary Figure 13. **Replicating results for Fig. 2 in the main text regarding gender disparity.** We repeat the analyses by selecting mid-career researchers with at least 20 papers. We find that the findings hold under the new productivity threshold. Two-sided t-test for comparisons. In (**a**, **b**, **d**, and **e**), shaded areas represent 95% confidence intervals.

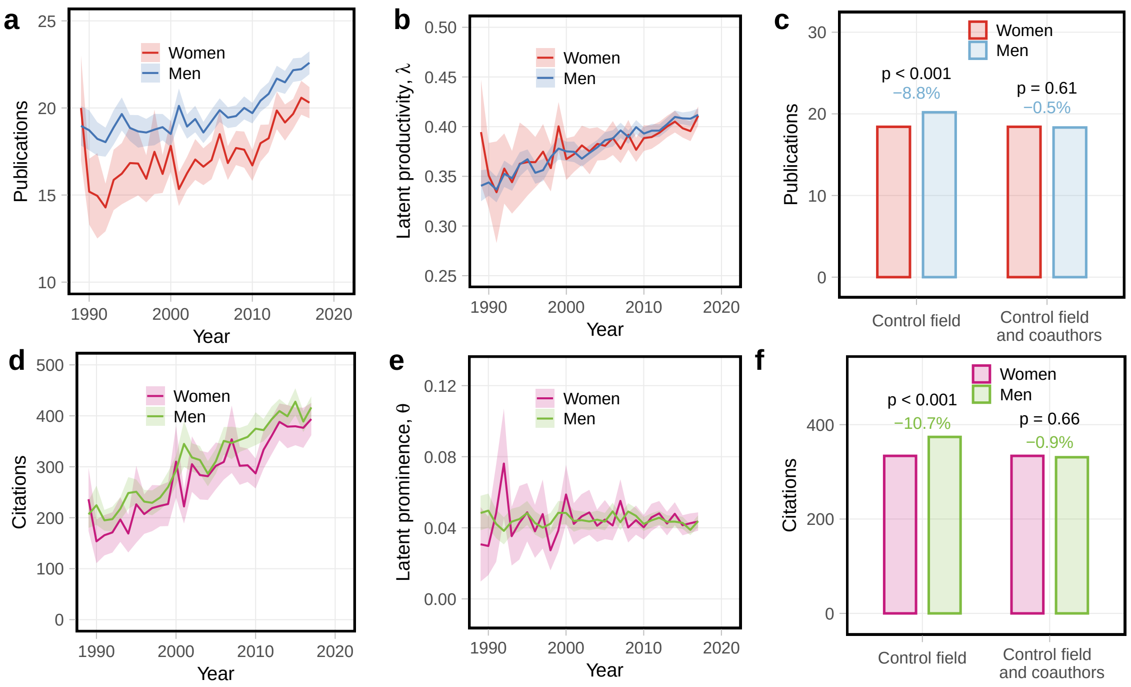

Supplementary Figure 14. **Replicating results for Fig. 2 in the main text regarding gender by sampling a tertile of researchers.** In this analysis, we randomly select a tertile of mid-career researchers, and find that our findings hold in the restricted sample of researchers. Two-sided t-test for comparisons. In (**a**, **b**, **d**, and **e**), shaded areas represent 95% confidence intervals.

Supplementary Note 6 Regression analyses

The regression analyses of individual researchers' latent parameters and the coauthorship network effects are shown in tables S5-S6. We present linear regression models to predict the productivity and logistic regression models to predict the prominence of mid-career researchers using their 15th year statistics. The covariates include the prestige score of the researcher's institution, researcher's latent parameter values of λ and θ, and network-based variables including the number of career coauthors, the number of high λ coauthors and the number of high θ coauthors. A researcher's λ has positive effects on both productivity and prominence, while the value of θ has negative effect on productivity and positive effect on prominence. The coauthorship network of a researcher, quantified by the coefficients of network-based variables, positively correlates with the

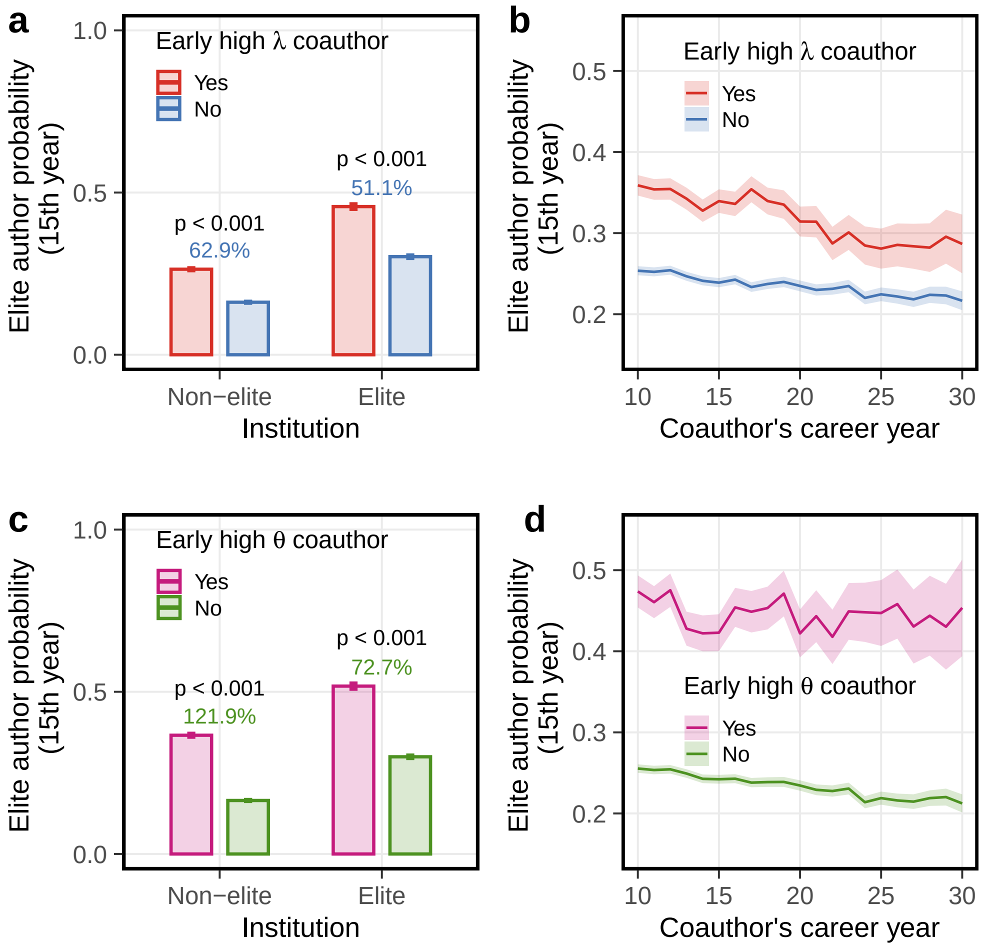

Supplementary Figure 15. **Replicating results for Fig. 3 in the main text regarding collaborations.** We use new thresholds to identify collaboration and aging effects. The selected senior collaborators are defined as those having at least 6 publications and at least 10 publishing career years by the time of relevant collaboration. Junior researchers with early high λ coauthors n for $\text{yes} = 41,469$; $\text{no} = 175,230$. Junior researchers with early high θ coauthors n for $\text{yes} = 19,534$; $\text{no} = 197,165$. Two-sided t-test for comparisons. Error bars in (**a** and **c**) indicate mean $\pm$ 1.96 SEM. In (**b** and **d**), solid lines indicate mean and shaded areas indicate 95% confidence intervals.

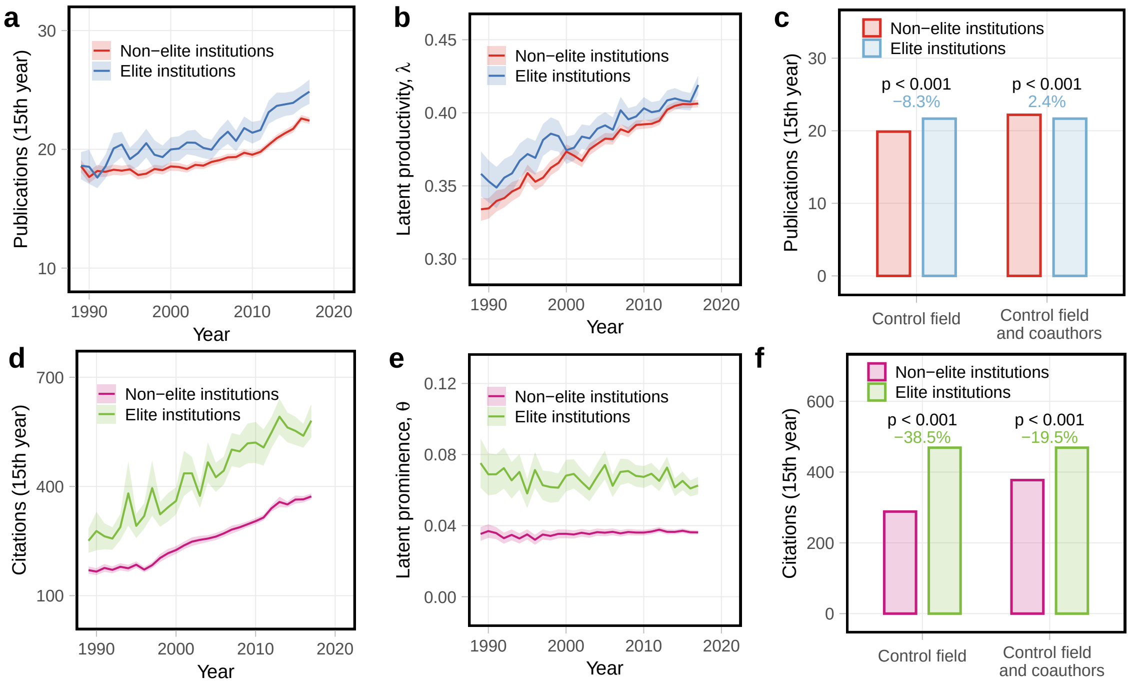

Supplementary Figure 16. **Replicating results for Fig. 4 in the main text regarding institutional prestige.** In this analysis, we select the top 20 research institutions as elite institutions, instead of top 10 institutions used in the main text. Our findings is consistent under the new threshold of elite institutions. Two-sided t-test for comparisons. In (**a**, **b**, **d**, and **e**), shaded areas represent 95% confidence intervals.

mid-career productivity and prominence. Taken together, we unveil the significant influence of a researcher's network on career development, shedding new light on the role of social networks in driving inequalities in science.

	Model 1	Model 2	Model 3
(Intercept)	11.872^{***}	-2.316^{***}	-2.109^{***}
	(0.089)	(0.039)	(0.040)
Institutional prestige	0.089^{***}		-0.050^{***}
	(0.003)		(0.001)
Author λ	21.123^{***}	9.698^{***}	9.710^{***}
	(0.212)	(0.082)	(0.081)
Author θ	-10.421^{***}	-4.391^{***}	-3.524^{***}
	(0.401)	(0.150)	(0.151)
Number of coauthors		1.323^{***}	1.330^{***}
		(0.002)	(0.002)
Number of high λ coauthors		1.038^{***}	1.033^{***}
		(0.003)	(0.003)
Number of high θ coauthors		0.166^{***}	0.182^{***}
		(0.006)	(0.006)
R^2	0.051	0.875	0.876
Adj. R^2	0.051	0.875	0.876
Num. obs.	198202	198202	198202

$^{***}p < 0.001$; $^{**}p < 0.01$; $^{*}p < 0.05$

Supplementary Table 5. Linear regression models to predict the number of papers (15th year). We analyze factors that affect the prominence of mid-career researchers using their 15th year statistics. The dependent variable is the number of total publications of mid-career researchers up to the 15th year. The covariates include the prestige score of the researcher's institution, researcher's latent parameter values of λ and θ, and network-based variables including the number of unique career-wise coauthors, the number of high λ coauthors and the number of high θ coauthors.

	Model 1	Model 2	Model 3
(Intercept)	-1.956^{***}	-4.033^{***}	-4.142^{***}
	(0.016)	(0.026)	(0.026)
Institutional prestige	0.029^{***}		0.020^{***}
	(0.001)		(0.001)
Author λ	1.169^{***}	0.689^{***}	0.695^{***}
	(0.037)	(0.047)	(0.047)
Author θ	12.194^{***}	14.655^{***}	14.383^{***}
	(0.090)	(0.104)	(0.105)
Number of coauthors		0.154^{***}	0.153^{***}
		(0.001)	(0.001)
Number of high λ coauthors		0.030^{***}	0.032^{***}
		(0.002)	(0.002)
Number of high θ coauthors		0.354^{***}	0.344^{***}
		(0.004)	(0.004)
AIC	209702.572	164067.646	163042.464
BIC	209743.360	164128.829	163113.843
Log Likelihood	-104847.286	-82027.823	-81514.232
Deviance	209694.572	164055.646	163028.464
Num. obs.	198202	198202	198202

$^{***}p < 0.001$; $^{**}p < 0.01$; $^{*}p < 0.05$

Supplementary Table 6. **Logistic regression models to predict if the researcher is a highly-cited author (15th year).** We analyze factors that affect the prominence of mid-career researchers using their 15th year statistics. The dependent variable uses a binary coding of whether the researcher is a top author in the 15th year or not. The covariates include the prestige score of the researcher's institution, researcher's latent parameter values of λ and θ, and network-based variables including the number of unique career-wise coauthors, the number of high λ coauthors and the number of high θ coauthors.

References

1. Ho, D. E., Imai, K., King, G. & Stuart, E. A. Matching as nonparametric preprocessing for reducing model dependence in parametric causal inference. *Political Analysis* **15**, 199–236 (2007).

2. Li, W., Aste, T., Caccioli, F. & Livan, G. Early coauthorship with top scientists predicts success in academic careers. *Nature Communications* **10**, 1–9 (2019).
