# Supplementary material for: Untangling the network effects of productivity and prominence among scientists
Source: Nat Commun. 2022 Aug 20;13:4907. doi: 10.1038/s41467-022-32604-6 (PMC9392727; doi:10.1038/s41467-022-32604-6)
Supplement: Supplementary file 2 — Reporting Summary [file 41467_2022_32604_MOESM2_ESM.pdf]

nature portfolio

Corresponding author(s): Weihua Li

Last updated by author(s): Aug 1, 2022

Reporting Summary

Nature Portfolio wishes to improve the reproducibility of the work that we publish. This form provides structure for consistency and transparency in reporting. For further information on Nature Portfolio policies, see our Editorial Policies and the Editorial Policy Checklist.

Statistics

For all statistical analyses, confirm that the following items are present in the figure legend, table legend, main text, or Methods section.

n/a	Confirmed	
☐	☒	The exact sample size (n) for each experimental group/condition, given as a discrete number and unit of measurement
☐	☒	A statement on whether measurements were taken from distinct samples or whether the same sample was measured repeatedly
☐	☒	The statistical test(s) used AND whether they are one- or two-sided<br>*Only common tests should be described solely by name; describe more complex techniques in the Methods section.*
☐	☒	A description of all covariates tested
☐	☒	A description of any assumptions or corrections, such as tests of normality and adjustment for multiple comparisons
☐	☒	A full description of the statistical parameters including central tendency (e.g. means) or other basic estimates (e.g. regression coefficient) AND variation (e.g. standard deviation) or associated estimates of uncertainty (e.g. confidence intervals)
☐	☒	For null hypothesis testing, the test statistic (e.g. F, t, r) with confidence intervals, effect sizes, degrees of freedom and P value noted<br>*Give P values as exact values whenever suitable.*
☒	☐	For Bayesian analysis, information on the choice of priors and Markov chain Monte Carlo settings
☒	☐	For hierarchical and complex designs, identification of the appropriate level for tests and full reporting of outcomes
☐	☒	Estimates of effect sizes (e.g. Cohen's d, Pearson's r), indicating how they were calculated

Our web collection on statistics for biologists contains articles on many of the points above.

Software and code

Policy information about availability of computer code

Data collection	We did not use any open source codes for data collection in this study.
Data analysis	Data were processed and analyzed using R version 4.1.1. In particular, we used R package data.table version 1.14.0 for processing and manipulating publication and citation data, R package CVXR version 1.0-10 to compute latent variable models, and R package ggplot2 version 3.3.5 for visualizations.

For manuscripts utilizing custom algorithms or software that are central to the research but not yet described in published literature, software must be made available to editors and reviewers. We strongly encourage code deposition in a community repository (e.g. GitHub). See the Nature Portfolio guidelines for submitting code & software for further information.

Data

Policy information about availability of data

All manuscripts must include a data availability statement. This statement should provide the following information, where applicable:

- Accession codes, unique identifiers, or web links for publicly available datasets
- A description of any restrictions on data availability
- For clinical datasets or third party data, please ensure that the statement adheres to our policy

The Microsoft Academic Graph data was obtained by following the guidelines at https://docs.microsoft.com/en-us/academic-services/graph/get-started-setup-provisioning.

Field-specific reporting

Please select the one below that is the best fit for your research. If you are not sure, read the appropriate sections before making your selection.

☐ Life sciences ☒ Behavioural & social sciences ☐ Ecological, evolutionary & environmental sciences

For a reference copy of the document with all sections, see nature.com/documents/nr-reporting-summary-flat.pdf

Behavioural & social sciences study design

All studies must disclose on these points even when the disclosure is negative.

Study description	This is a quantitative study of network models to estimate the latent productivity and prominence of researchers based on pre-existing datasets.
Research sample	We selected six representative STEM fields including biology, chemistry, computer science, mathematics, medicine, and physics using using 20 million publication data from the Microsoft Academic Graph.
Sampling strategy	No statistical methods were used to predetermine sample size.
Data collection	This study is based on pre-existing datasets.
Timing	The publication data used in this study were downloaded on February 15th, 2020 from the Microsoft Azure server.
Data exclusions	No data were excluded from the analysis. Selection criteria within the dataset are described in the Methods section.
Non-participation	No dropout participants.
Randomization	This is a data-driven study, not a randomized experiment.

Reporting for specific materials, systems and methods

We require information from authors about some types of materials, experimental systems and methods used in many studies. Here, indicate whether each material, system or method listed is relevant to your study. If you are not sure if a list item applies to your research, read the appropriate section before selecting a response.

Materials & experimental systems

n/a	Involved in the study	
☒	☐	Antibodies
☒	☐	Eukaryotic cell lines
☒	☐	Palaeontology and archaeology
☒	☐	Animals and other organisms
☒	☐	Human research participants
☒	☐	Clinical data
☒	☐	Dual use research of concern

Methods

n/a	Involved in the study	
☒	☐	ChIP-seq
☒	☐	Flow cytometry
☒	☐	MRI-based neuroimaging
